# Identification of a Novel Homozygous Mutation in *PRDM12* Gene in a Patient with Hereditary Sensory and Autonomic Neuropathy Type VIII

**DOI:** 10.34172/aim.2024.32

**Published:** 2024-04-01

**Authors:** Amir Hossein Ebrahimi, Manzar Bolhassani, Mohammad Reza Zarei, Matin Heidari, Amin ArdeshirDavani, Amir Hosein Mehrtash, Zahra Shiri, Masoud Heidari, Morteza Soleyman-Nejad, Mohammad Hossein Taskhiri, Arefeh Norouzbeigi, Mansour Heidari

**Affiliations:** ^1^Ariagene Medical Genetics Laboratory, Qom, Iran; ^2^Department of Cellular and Molecular Genetics, Islamic Azad University Qom Branch, Qom, Iran; ^3^Department of Cellular and Molecular Genetics, Islamic Azad University North Tehran Branch, Tehran, Iran; ^4^Cimorgh Medical IT Solutions, Tehran Iran; ^5^Department of Medical Genetics, Tehran University of Medical Sciences (TUMS), Tehran, Iran

**Keywords:** Duplication mutation, HSAN-VIII, *PRDM12* gene, Whole exome sequencing

## Abstract

Hereditary sensory autonomic neuropathy type VIII (HSAN-VIII) is a rare genetic disease that occurs due to mutations in the *PRDM12* gene. Here, we describe a novel homozygous mutation c.826_840dupTGCAACCGCCGCTTC (p.Cys276_Phe280dup) on exon 5 in the *PRDM12* gene identified by WES and confirmed using Sanger sequencing method.

## Introduction

 HSAN-VIII (OMIM 616488) is a neurological disorder with an autosomal recessive inheritance that occurs with homogenous pathogenic mutations in the *PRDM12* gene located on chromosome 9q34.^1^ Pathogenic variants on *NGF*, *SCN9A*, *NTRK1*, *IKBKAP*, *KIF1A*, *RETREG1*, and *WNK1* genes are causes of HSAN-VIII. The main characteristic of this disorder is congenital insensitivity to pain that is associated with ulceration in the tongue, lips, fingers, and other organs.^2,3^ Due to the wide range of abnormalities and overlap of clinical symptoms that are associated with different pathogenic mutations on various genes, diagnosis of HSAN subtypes is difficult, and molecular genetic analysis can be helpful.

 The present case report describes the exome analysis of an Iranian patient with HSAN-VIII born to consanguineous parents and the identification of a novel, likely pathogenic sequence variant in the *PRDM12* gene segregating with the disease. Our data provide a diagnostic strategy for genetic causes by whole exome sequencing (WES).

## Case Report

**Figure 1:**
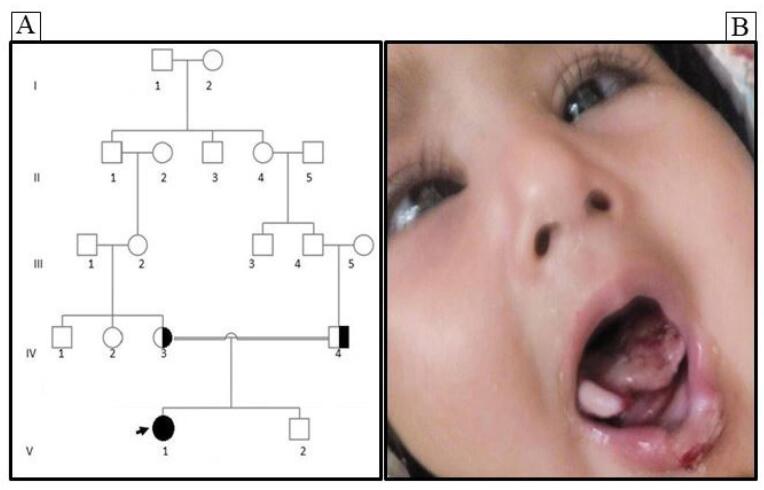
Pedigree Analysis and Clinical Features of an Iranian Family with HSAN-VIII. A) The pedigree of the family represents an autosomal recessive pattern of inheritance. B) Phenotype demonstrating HSAN-VIII with visual problems, and tongue and lip defects. A filled symbol represents an HSAN-VIII patient, open symbols reveal individuals without clinical HSAN-VIII while the half-filled symbols show heterozygous individuals (arrow indicates the proband)

 An 11-month-old Iranian girl born to consanguineous parents (Iranian Persian family), initially referred to the Medical Genetics Laboratory (Aria Gene Lab, Qom, Iran) was evaluated in October 2020 (Figure 1A). Numerous traumas without any sign of discomfort were found in the patient. She had unusual lesions and injuries, including oral and tongue injuries. Eye problems such as nystagmus were observed as the first clinical sign of abnormal vision (Figure 1B). In addition, dry skin with no sweat and recurrent unexplained fever was observed. General examination indicated evidence of several scars and non-tender ulcers in limbs and face. Moreover, neurologic examination revealed loss of reflexes with the general absence of pain and temperature perception. The parents of the proband were informed about this study and signed informed consent.

 To embark on the exome sequencing journey, the highly sophisticated SureSelect Human All Exon V7 enrichment Kit (Agilent Technologies, Santa Clara, California, USA) was employed for precise and comprehensive exome sequence capture. This cutting-edge kit ensures the targeted enrichment of all exonic regions, allowing for thorough exploration of the protein-coding regions of the genome. Subsequently, the captured library, representing the exonic content of the genome, was subjected to 2 × 150 paired-end sequencing using the NovaSeq 6000 Sequencer (Illumina, San Diego, California, USA).^4^ The utilization of paired-end sequencing enhances the accuracy and completeness of the data by providing sequencing reads from both ends of the DNA fragments, thereby enabling a more comprehensive analysis of genetic variations, including insertions, deletions, and structural rearrangements.

 This meticulous exome sequencing approach, integrating advanced genomic technologies and high-throughput sequencing platforms, ensures comprehensive exploration of the coding regions of the genome, providing valuable insight into the genetic landscape associated with the patient’s condition.

 The bioinformatics analysis of the acquired data was performed through an internally developed and clinically validated Genome Analysis Platform (GenAP) pipeline, a proprietary system provided by Cimorgh Medical IT Solutions in Tehran, Iran. Leveraging cutting-edge technologies, the alignment and identification of genetic variants were accomplished using a tandem approach involving the Burrows-Wheeler Aligner (BWA) and Google DeepVariant software. This dual-method strategy ensures comprehensive and accurate identification of genetic variants, enhancing the reliability of the subsequent analyses.

 To refine the dataset, variants situated outside predefined target regions and those within regions with coverage below 10x were meticulously filtered out, resulting in a focused dataset for further analysis. The filtered variant call format (VCF) file then underwent a detailed annotation process using the GenAP annotation pipeline, which integrates advanced algorithms and curated databases to enrich the variant information.

 A noteworthy feature of the GenAP pipeline is its automated evaluation of 18 out of the 28 ACMG/AMP criteria, streamlining the variant classification process. However, for criteria labelled as “MANUAL,” an automated trigger is not activated. In such instances, additional information sourced from the patient’s clinical records or co-segregation studies is imperative to inform the variant classification decision. This meticulous approach ensures a nuanced and context-aware assessment, aligning with the high standards set by the ACMG guidelines. The integration of GenAP into the analytical workflow exemplifies a commitment to precision and thoroughness in variant calling, annotation, and classification processes, laying a robust foundation for accurate genetic analysis.

 The variant filtration strategy was initiated by excluding variants with an allelic frequency higher than 0.01 in extensive normal population databases, including gnomAD, ExAC, and an in-house variant frequency dataset derived from an extensive cohort of over 12 000 samples representative of the Iranian population. Subsequently, the integration of homozygous variants based on familial pedigree data further refined the candidate variants. To prioritize variants associated with neuropathy disorders, a meticulously curated gene list was curated by the Genomics England PanelApp (Hereditary neuropathy ID: 85), leading to the identification of a potential causative variant within the *PRDM12* gene.^5^

 Following the stringent guidelines set by the American College of Medical Genetics and Genomics (ACMG), the allelic frequency of the c.826_840dupTGCAACCGCCGCTTC (p.Cys276_Phe280dup) variant in exon 5 of the *PRDM12* gene was observed to be notably low in the normal population (PM2). Computational analysis utilizing the PaPI tool predicted a deleterious impact for this variant, assigning it a high score of 0.904 (PP3). Notably, missense and in-frame shift variants within this specific region of *PRDM12*, known as the zinc finger domain ‘C2H2-type 2,’ have been documented as likely pathogenic and uncertain of significance, respectively, with a conspicuous absence of benign variants. (PM1). Protein coding length changes as a result of in-frame variant in gene *PRDM12*, and this variant is not located in a repeat region (PM4). Collectively, this variant has been identified as likely pathogenic according to the ACMG guideline.

 Crucially, the identified in-frame variant does not locate with any repeat regions, enhancing its significance. As a result of this comprehensive analysis, taking into account allelic frequency, computational predictions, clinical significance and the location within a region associated with known pathogenic variants, the variant has been judiciously classified as likely pathogenic. This meticulous approach exemplifies a commitment to precision in variant prioritization, aligning with the highest standards of genomic interpretation and facilitating informed decision-making in the context of genetic diagnostics. Finally, the detected mutation was confirmed using Sanger sequencing.

**Figure 2:**
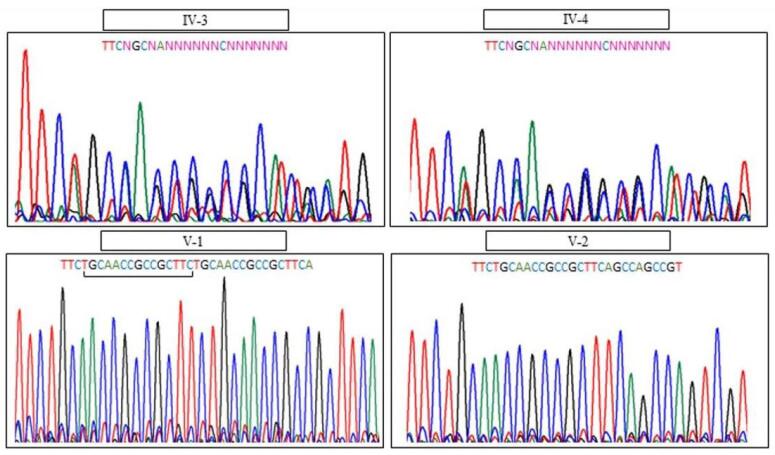
Molecular Study of the HSAN-VIII Disease. A novel homozygous mutation c.826_840dupTGCAACCGCCGCTTC (p. Cys276_Phe280dup) on exon 5 in the *PRDM12* gene was identified by WES. Sanger sequencing showed familial segregation of the mutation which is consistent with autosomal recessive inheritance. The arrow shows the duplication region

 Both parents were heterozygote for PRDM12 mutation. Therefore, PND was performed in next pregnancy by evaluating the fetal DNA through amniocentesis and the boy was normal homozygote for PRDM12 gene mutation (Figure 2).

## Discussion

 The five main characteristics of patients with HSAN-VIII are recurrent infections of the skin and bones, absence of corneal reflexes, altered formation of sweat and tear, self-mutilation behavior, and insensitivity to pain and temperature.^6^ Loss of pain perception is due to defects in the development of sensory neurons as a result of mutations in the *PRDM12* gene. Therefore, patients with *PRDM12* mutations are insensitive to acute pain *and* temperature.^7,8^

 Here, we used WES for detection of mutations in an 11-month-old Iranian female with suspicious clinical symptoms of HSAN. In addition, confirmation and validation of the detected mutation were performed by the Sanger sequencing method. We detected a novel duplication mutation in the *PRDM12* gene in homozygote status by WES. This mutation was in a heterozygous state in the family of the patient. Knowledge of underlying mechanisms in the destruction of nerve fibers or pain receptors by mutations in the *PRDM12 *gene can be beneficial in developing novel therapeutic strategies for patients with HSAN-VIII. Furthermore, expanding our knowledge of epigenetic and genetic factors that are involved in pain sensation creates new fields for therapeutic intervention in patients with neuropathic sensory abnormalities.

 Collectively, our results demonstrated that patients with mutations in the *PDRM12* gene can be clinically diagnosed through pain and temperature insensitivity, impaired sweat and tear production, absence of corneal reflex, and multiple oral and tongue ulcers. However, the final and accurate diagnosis of HSAN-VIII is confirmed by molecular sequencing tests such as WES. We showed more details for the application of WES as a powerful and cost-effective tool for molecular diagnosis of heterogeneous disorders like HSAN-VIII. However, further studies are required to understand the genotype-phenotype association in patients carrying duplication mutations in the *PRDM12* gene.
